# Effects of Fermentation Compound Chinese Herbal Medicine on the Reproductive Performance, Immune and Antioxidant Status, and Colostrum Metabolites of Ningxiang Sows During the Lactation Period

**DOI:** 10.3390/ani16020167

**Published:** 2026-01-07

**Authors:** Qingtai Zhang, Haibo Huang, Xinhao Song, Weiguang Yang, Rejun Fang, Chengkun Fang

**Affiliations:** 1 College of Animal Science, Hunan Agricultural University, Changsha 410128, China; zhangqt00@126.com (Q.Z.); haibo_huang1024@163.com (H.H.); 18600132950@163.com (X.S.); yangweiguang1@stu.gdou.edu.cn (W.Y.); 2 Hunan Engineering Research Center of Intelligent Animal Husbandry, Changsha 410128, China

**Keywords:** fermented Chinese herbal medicine, reproductive performance, milk quality, suckling piglets, Ningxiang sow

## Abstract

The present study aimed to evaluate the effects of fermented compound Chinese herbal medicine (FCHM, *Vaccaria segetali*, *Tetrapanax papyriferus*, *Ligusticum chuanxiong* Hort and *Rhaponticum uniflorum*) on the reproductive performance of Ningxiang sows. A total of 30 Ningxiang sows were randomly assigned to the control group (CON), CHM group, and FCHM group. The results indicated that dietary CHMs and FCHM supplementation significantly increased (*p* < 0.05) the milk production, feed intake, reproductive performance protein content, and lactose content in the milk of sows and significantly decreased (*p* < 0.05) the number of somatic cells in colostrum and in the number of low-birthweight piglets. Dietary FCHM supplementation significantly increased (*p* < 0.05) the number of healthy piglets, birth litter weight, colostrum fat content, and feed intake. Moreover, malondialdehyde, porcine interleukin-1α, and porcine interleukin-6 in sow serum were decreased (*p* < 0.05), and the activity of superoxide dismutase, total antioxidant capacity, immunoglobulin, prolactin and progesterone levels (*p* < 0.05) were increased, with FCHM supplementation. The colostrum metabolomics analyses showed that FCHM significantly enriched the oxytocin signaling pathway, calcium signaling pathway, and pathways associated with milk composition synthesis. In conclusion, supplementing with FCHM improved the reproductive performance and milk metabolic biomarkers of sows and may serve as an effective feed additive to improve productivity.

## Simple Summary

Low lactation performance and postpartum inflammation in Ningxiang sows are major problems in sow farming, negatively affecting piglet growth and sow welfare. Chinese herbal medicines (CHMs) mainly come from plants, animals, and minerals that have been reported to possess multiple biological functions, including antioxidant, immunomodulatory, anti-inflammatory, and anti-microbial activities. This study explores the effect of fermented CHMs on Ningxiang sow reproductive performance and health improvement. The present study showed that fermented CHMs can significantly increase the milk yield and overall immunity of sows, improve the milk quality of sows, and improve the growth performance and immune level of piglets. This study can provide a reference for the application of fermented CHMs as an alternative to antibiotics in sows.

## 1. Introduction

Ningxiang pigs are native to Liushahe, Caochong, and other areas within Ningxiang County, Hunan Province, boasting a history of over 1000 years in China. Ningxiang pigs are early-maturing and exhibit high fat deposition, making them a representative semi-lard-type breed among Chinese indigenous pigs raised for both meat and fat. They are known for superior meat quality, dietary adaptability to roughage, and robust stress resistance [1,2]. However, their reproductive efficiency and growth rate are lower than those of crossbred lines such as Duroc × Landrace × Yorkshire [3]. In intensive farming, the overuse of antibiotics has led to an increase in drug-resistant bacterial strains, exacerbating the incidence of endometritis and mastitis; milk secreted by sows with metritis and mastitis often contains harmful microbes and pro-inflammatory mediators.

Chinese herbal medicines (CHMs), long treasured as the cornerstone of Chinese ethnopharmacology, are increasingly recognized as a promising antibiotic alternatives, owing to their multi-target regulatory mechanisms, minimal residue profiles, and low propensity to induce resistance. CHMs or extracts are frequently incorporated into ruminant and porcine rations to enhance lactational performance in dairy cow and sows [4,5] because CHMs contain bioactive components that possess antibacterial, anti-inflammatory, anti-oxidative, and immune-enhancing properties. Supplementation with *Vaccaria segetalis* alone has demonstrated the ability to enhance lactation performance in sows [6]. *Tetrapanax papyriferus* can promote the secretion of lactation-related hormones [7]. The volatile oil from *Ligusticum chuanxiong* Hort can enhance antioxidant capacity, improve lactation performance, and elevate milk quality [8]. *Rhaponticum uniflorum* possesses physiological activities such as immune stimulation and anti-inflammatory effects. Moreover, its ethanol extract has been shown to improve milk quality [9,10]. Herbs exhibit complementary and synergistic roles, with principal, secondary constituents that can be strategically combined [11]. Multi-herb formulations consistently surpass equivalent doses of single herbs, highlighting the centrality of synergy in herbal therapeutics [12,13]. *Limosilactobacillus fermentum*, a lactic acid bacterium used for fermenting macadamia nut pericarp, significantly increases total polyphenol and total flavonoid contents while preserving antioxidant activity [14,15]. This study investigates an innovative approach to enhancing the reproductive performance of Ningxiang pigs through the application of fermented Chinese herbal medicine (FCHM), integrating microbial fermentation technology with traditional Chinese veterinary medicine principles, specifically utilizing *Limosilactobacillus fermentum* as the fermentative agent.

## 2. Materials and Methods

### 2.1. Preparation of Chinese Herbal Medicine Fermentation

According to the classical Chinese pharmacopeia (State Pharmacopoeia Commission of the PRC, 2020) [16], the CHMs used in this study were prepared from 4 Chinese herbs, including *Vaccaria segetalis*, *Tetrapanax papyriferus*, *Rhaponticum uniflorum*, and *Ligusticum chuanxiong* Hort (proportion of four herbs were 6:2:2:1) by weight; all herbal materials were purchased from Anhui, China. Impurities were removed the herbs before they were mixed thoroughly and crumbled, rinsed with water, naturally air-dried to a moisture content of <11%, and crushed and sieved through a 40-mesh sieve.

The FCHM was prepared as follows. A substrate composed of 70% compound CHM powder, 25% soybean meal, and 5% calcium carbonate (*w*/*w*) was thoroughly mixed and sterilized by autoclaving at 121 °C, 0.15 MPa for 30 min. A suspension of *Limosilactobacillus fermentum ZC529* (CCTCC M20222085) was adjusted to 1 × 10^8^ CFU/mL and used to inoculate the cooled substrate at 4% (mL per 100 g). The moisture content was brought to 40%, and the mixture was incubated anaerobically at 37 °C for 7 days in sealed bags fitted with one-way valves. The final product was low-temperature dried, packaged, and stored until use. The strain and any derivative sub-clones thereof were deposited at the China Center for Type Culture Collection, Wuhan, China. The process is protected under Chinese patent no. CN119896281A.

### 2.2. Sows, Experiment Design, Diets, and Management

The present study was conducted at a farm in Hunan province, China, which is characterized as having a subtropical monsoon climate. A total of 30 Ningxiang pregnant sows with similar parities (2–4 parity) and pregnancies (100 days) were randomly allocated into the control group (CON; sows fed a basal diet), the CHM group (CHM; sows fed a basal diet with 2% Chinese herbal medicine replacing corn) and FCHM group (FCHM; sows fed a basal diet with 2% fermented Chinese herbal medicine replacing corn) with 10 replicates each. The experiment consisted of a 7-day pre-feeding period and a 28-day experimental period. The experimental period spanned from 7 days prepartum to 21 days of lactation. The basal diet (Table 1) was formulated to meet or exceed the requirements of pregnant sows, as outlined by the National Research Council (NRC, 2012) [17].

Standard farm biosecurity and disinfection protocols were strictly maintained throughout the experimental period. The sows were fed twice a day at 6:00 a.m. and 4:00 p.m., with free access to feed and water. The feed intake of the sows was recorded every day. Feed residues were monitored and quantified daily; any remaining feed was collected, weighed, and recorded, when present. Consistent with experimental designs evaluating phytase in peripartum sows [18], ad libitum feeding was implemented to eliminate restriction bias on a voluntary intake assessment. Daily postpartum monitoring included the occurrence of watery feces in pens, and fecal adherence to piglet perianal regions.

### 2.3. Sample Collection

On the day of farrowing, 8 sows per group were randomly selected for marginal ear vein blood collection. On day 28, 8 litters per group were selected, with 1 piglet randomly chosen per litter for 10 mL cranial vena cava blood sampling using standard vacutainers. Blood samples were clotted for 30 min, centrifuged at 4000× *g* for 15 min, with serum aliquoted into 1.5 mL microcentrifuge tubes, and stored at −20 °C. Within 4 h postpartum, 20 mL colostrum was collected by pooling samples from anterior, middle, and posterior mammary glands, and stored at −80 °C.

### 2.4. Feed Sample Collection and Chemical Analysis

During the experiment, diet samples (100 g) from different treatment groups were collected and kept at −20 °C until analysis. The crude protein content in both herbal additives and basal diets was determined according to GB/T 6432-2018 [19]. The ether extract was analyzed following GB/T 6433-2006 [20]. The crude fiber content was measured by GB/T 6434-2006 [21]. Ash content was determined by GB/T 6438-2007 [22]. The calcium content was determined according to the national standard GB/T 6436-2018 [23]. The total phosphorus content was determined according to the national standard GB/T 6437-2018 [24]. Total polysaccharide content was determined by the anthrone–sulfuric acid method. Total flavonoids were quantified using the sodium nitrite–aluminum nitrate method. Total alkaloids were measured by acidic dye colorimetry. Total saponins were analyzed via vanillin–perchloric acid assay.

### 2.5. Reproductive Performance

At farrowing, the number of total piglets born, the number of born-alive piglets, the number of low-birthweight piglets, the number of high-birthweight piglets, the number of stillborn piglets, and the total born process time were recorded. The total litter birthweight and average birthweight were calculated (weighed within 12 h after birth). Piglets with birthweights ≤ 0.8 kg were defined as low-birthweight piglets; those with birthweights > 0.8 kg were defined as high-birthweight piglets.

The average born process was calculated by the following formula: the average born process time (min) = total born process time/number of total born piglets. The milk production was measured as the method described by [25]. The formula is as follows: lactation yield (kg) = piglet ADG × number of piglets × days of lactation × 4.

### 2.6. Growth Performance

The weaning litter weight and the number of weaned piglets were recorded for the 28-day experiment. The rate of survival and diarrhea was calculated by the following formula: survival rate (%) = number of weaned piglets/number of liveborn piglets × 100; diarrhea rate (%) = (number of diarrheic piglets × number of diarrhea days)/(total number of suckling piglets × lactation period days) × 100; and weaning individual mean weight = litter weight of weaned piglets/number of weaned piglets × 100.

### 2.7. Serum Biochemical Indices

The contents of prolactin (PRL), estrogen (E2), progesterone (Prog), immunoglobulin A (IgA), immunoglobulin M (IgM), immunoglobulin G (IgG), interleukin-1α (IL-1α), interleukin-6 (IL-6), and serum tumor necrosis factor (TNF-a), were determined using enzyme-linked immunosorbent assay (ELISA) kits, following the protocol provided by the manufacturer. (Wuhan ELK Biotechnology Co., Ltd., Wuhan, China).

The malondialdehyde (MDA) content, reduced glutathione peroxidase (GSH-Px) activity, superoxide dismutase (SOD) activity, and total antioxidant capacity (T-AOC) were determined using commercial assay kits (Suzhou Grace Biotechnology Co., Ltd., Suzhou, China) according to the manufacturer’s instructions.

### 2.8. Colostrum Ingredients

The ingredients of the colostrum were evaluated for various parameters, such as protein percentage, fat percentage, lactose percentage, urea nitrogen, content, non-milk fat solid content, and total dry matter, using a fully automated milk analyzer (MilkoScan™ FT+200, FOSS, Hilleroed, Denmark). Concurrently, the somatic cell count was determined with a cell analyzer (Type 79910, Fossomatic FC, FOSS, Hilleroed, Denmark), strictly adhering to the manufacturer’s instructions.

### 2.9. Non-Targeted Metabolism of Colostrum

Samples were retrieved from a −80 °C freezer and placed on dry ice. One hundred μL of colostrum sample was aspirated and combined with 400 μL of pre-cooled extraction solution (−40 °C; methanol: acetonitrile = 3:1, *v*/*v*). The mixture was vortex-mixed for 5 min, followed by sonication for 15 min, and incubated at 4 °C for 1 h. After incubation, samples were centrifuged at 12,000 rpm for 15 min (4 °C). An equal volume of supernatant was transferred and vacuum-concentrated to dryness. The dried metabolites were reconstituted in 50 μL of 50% aqueous methanol (methanol: water = 1:1, *v*/*v*), followed by vortex-mixing for 3 min at 4 °C (2000 rpm) and centrifugation at 12,000 rpm for 15 min (4 °C). The resulting supernatant was transferred to a 2 mL injection vial; the procedure was entrusted to Vicbio Biotechnology Co., Ltd. (Beijing, China).

### 2.10. Statistical Analysis

All statistical analyses were conducted using the SPSS 26.0 software (SPSS Inc., Chicago, IL, USA). One-way ANOVA by Duncan’s multiple-range test was used to analyze the differences among CON, CHM and FCHM. Results were expressed as means with SEM; significance was set at *p* < 0.05.

## 3. Results

### 3.1. Changes in Nutrients and Active Components

The FCHM exhibited a uniform brown color with a mild sour aroma and distinct herbal fragrance after fermentation. The product displayed a loose, non-caking texture with well-defined physical structure and no visible mold growth. As presented in Table 2, significant improvements in nutritional and bioactive components were observed: crude protein contents were increased by 13.55%, crude fiber content rose by 12.32%, total flavonoid contents were elevated by 23%, and total polysaccharide contents were enhanced by 34% compared to the unfermented control.

### 3.2. Reproductive Performance

As shown in Table 3, dietary supplementation with FCHM significantly improved sow reproductive performance compared to the CON group. Both the CHM and FCHM groups had fewer low-birthweight piglets (*p* < 0.05), while demonstrating increased sow weight loss, average piglet birthweight and milk yield (*p* < 0.05). Notably, the FCHM group outperformed the CHM group, showing a significant increase in total litter birthweight (*p* < 0.05).

### 3.3. Feed Intake

As presented in Table 4, dietary supplementation with FCHM significantly increased feed intake during both the gestation and lactation periods compared to the CON group (*p* < 0.05).

### 3.4. Growth Performance

As shown in Table 5, supplementation with both CHM and FCHM significantly improved piglet growth performance compared to the CON group (*p* < 0.05).

### 3.5. Colostrum Components

The effects of FCHM supplements in the feed on the colostrum composition in sows are shown in Table 6. Compared with the CON group, both the CHM and FCHM groups exhibited significantly reduced somatic cell counts in colostrum (*p* < 0.05), while milk protein percentage and lactose content were significantly increased (*p* < 0.05), the FCHM group demonstrated significantly higher milk fat rate and UN contents (*p* < 0.05).

### 3.6. Serum Biochemical Indicators

The effects of dietary FCHM supplementation on serum biochemical indices in sows and piglets are shown in Table 7. Compared with the CON group, dietary supplementation with CHM and FCHM significantly decreased (*p* < 0.05) the concentrations of MDA, IL-1α, IL-6, and E2, while increasing SOD activity, IgG, and Prog levels (*p* < 0.05). Compared with both the CHM and CON groups, the FCHM group exhibited further significant increases (*p* < 0.05) in T-AOC, IgM, and PRL, and also showed elevated IgA levels compared with the CON group (*p* < 0.05). In piglets, only IgG levels showed an increasing trend (0.05 < *p* < 0.10).

### 3.7. Non-Target Differential Metabolites of Colostrum

**Figure 1:**
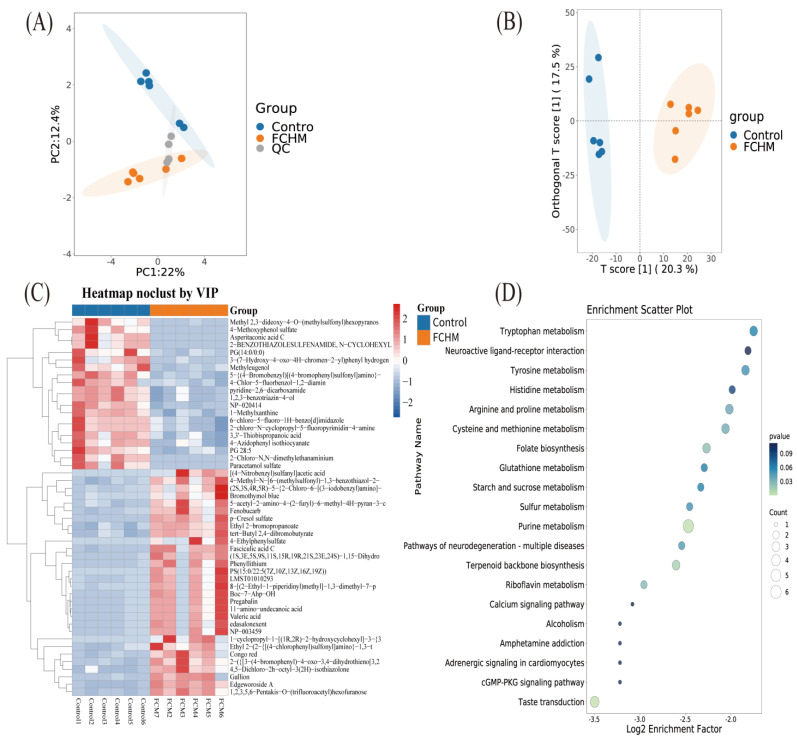
Effects of dietary supplementation with FCHM on differential metabolites in sow colostrum: (**A**) quality-control–principal-component analysis (QC–PCA) plot; (**B**) orthogonal partial least-squares–discriminant analysis (OPLS–DA); (**C**) heatmap; and (**D**) bubble plot.

Ultra-high-performance liquid chromatography–mass spectrometry (UHPLC–MS) profiling identified 3446 metabolites in the colostrum of Ningxiang sows. In the quality-control–principal-component analysis (QC–PCA) plot, the QC samples formed a tight cluster (Figure 1A), confirming high analytical reproducibility. Orthogonal partial least-squares–discriminant analysis (OPLS-DA) further revealed a clear separation of colostrum metabolic profiles between the FCHM and CON groups (Figure 1B). Model validation metrics (R^2^X = 0.391, R^2^Y = 0.991, Q^2^ = 0.762) satisfied the acceptance criteria, indicating a robust and predictive model suitable for downstream differential metabolite analysis. A total of 498 differentially expressed metabolites were identified between the CON and FCHM groups, including 217 upregulated and 287 downregulated metabolites. A heatmap was generated for the top 50 differential metabolites ranked by variable importance in the projection (VIP) scores (Figure 1C), and a bubble plot was constructed to illustrate the enriched metabolic pathways of significantly altered metabolites (Figure 1D). The metabolic pathway analysis indicated that the differences in colostrum composition between the FCHM and CON groups primarily involved amino acid metabolism, cofactor and vitamin metabolism, and carbohydrate metabolism. Compared to CON, the FCHM group exhibited marked alterations in key KEGG pathways, including OT and PRL signaling, calcium signaling pathway, galactose/starch/sucrose metabolism, aminoacyl-tRNA biosynthesis, fatty acid biosynthesis, pantothenate and CoA biosynthesis, oxidative phosphorylation, and ABC transporters.

The effects of dietary supplementation with FCHM on differential metabolites in sow colostrum are presented in Table 8. Compared with the control group, the FCHM group showed significant upregulation (*p* < 0.05) of metabolites including p-cresol sulfate, edgeworoside A, ethyl 2-bromopropanoate, fasciculic acid C, tert-butyl 2,4-dibromobutyrate, pregabalin, 4-ethylphenylsulfate, edasalonexent, 11-amino-undecanoic acid, valeric acid and phenyllithium. Conversely, metabolites, such as 2-Chloro-N,N-dimethylethanaminium, 4-methoxyphenol sulfate, paracetamol sulfate, asperitaconic acid C, 1-methylxanthine, N-Cyclohexyl-2-benzothiazolesulfenamide, pyridine-2,6-dicarboxamide, methyleugenol, and PG 28:5. were significantly downregulated (*p* < 0.05).

## 4. Discussion

The flavonoid glycosides in *Vaccaria segetalis* have been demonstrated to upregulate hexokinase II expression in rat skeletal muscle, thereby enhancing glucose utilization [26]. Similarly, the flavonoid glycoside tilianin was found to ameliorate oxidative stress and regulate energy metabolism disorders in a murine model of myocardial ischemia/reperfusion injury [27]. While CHM and FCHM supplementation increased sow weight loss compared to the control group, this appears to reflect enhanced milk production rather than compromised health. The elevated serum prolactin and improved piglet growth performance observed in our study suggest greater metabolic demand for lactogenesis. We propose that the herbal formulations increased nutrient partitioning toward milk synthesis, resulting in greater mobilization of maternal body reserves. This interpretation is supported by the fact that, despite higher weight loss, sows in the FCHM group showed improved immune markers and no signs of metabolic stress. Neonatal piglets rely entirely on the nutrients and energy in sow’s milk for thermoregulation and growth. Enhanced milk production can shorten weaning time, increase weaning weight, and accelerate production cycles. Dibutyl phthalate from *Vaccaria segetalis* exhibits estrogen-like activity, upregulating lactation-related genes and promoting mammary epithelial cell proliferation [28]. *Vaccaria segetalis* flavonoid glycosides directly stimulate mammary epithelial cell proliferation [29]. The rise in luminal lactose elevates the osmotic pressure within the alveolar lumen, driving the passive influx of water and thereby augmenting milk yield. *Ligustilide* from *Ligusticum chuanxiong* improves peripheral circulation and mammary blood supply [30], ensuring optimal nutrient delivery for milk synthesis. The synergistic actions of these herbs collectively potentiate milk production, explaining the significantly higher lactation performance observed in the FCHM group.

The peripartum period induces significant immunological and metabolic alterations in sows, often resulting in reduced feed intake and decreased milk production. In the present study, sows receiving the FCHM supplementation demonstrated significantly enhanced feed intake during the first and third weeks postpartum. This improvement is attributed to the fermentation process, which modified the herbal formulation’s sensory characteristics by reducing characteristic bitter compounds, thereby enhancing palatability and voluntary intake [31]. CHM with *Limosilactobacillus fermentum ZC529* significantly enhanced its nutritional quality, as evidenced by increased crude protein, total flavonoids, and polysaccharide content. Importantly, the fermentation process also modified the herbal formulation’s sensory characteristics by reducing its characteristic bitter taste, thereby improving overall palatability and quality.

The growth performance of suckling piglets is comprehensively influenced by multiple factors, including colostrum composition, milk yield, and litter birthweight. In this study, piglets from the FCHM group showed significantly higher weaning litter weight and average individual weight compared to the CON group, which can be attributed to several key improvements: the FCHM group exhibited enhanced milk production and superior nutritional composition, leading to better nutrient intake. Simultaneously, the boosted maternal immunity benefited fetal development in utero; stronger piglet immunity directly supported weight gain during lactation [32]. While fermentation clearly enhanced bioavailability, the lack of additional growth benefits in piglets from FCHM versus CHM likely reflects several factors. First, the non-fermented CHM already contained sufficient bioactive compounds to support maximal piglet growth in this study, creating a plateau effect where fermentation could not provide further growth advantages. Second, growth performance may be less sensitive than biochemical markers to modest improvements in bioavailability. The sow health benefits we observed primarily support piglet survival and immune competence rather than directly accelerating growth rate.

Colostrum is the first mammary secretion produced within 24 h postpartum. The amount of colostrum intake shows a significant correlation with piglet mortality [33]. Key nutritional indicators, including milk fat percentage, milk protein, and lactose content, serve as crucial parameters for evaluating milk quality. The bioactive components in *Vaccaria segetalis* modulate the activity of key enzymes involved in glucose metabolism within mammary epithelial cells, maintaining high cellular energy levels that facilitate the biosynthesis of lactose, milk proteins, and milk fat from glucose [34]. *Vaccaria* effectively increased the milk production, significantly promoted the PRL secretion expression in lactation-insufficiency model rats [35]. Using a mammary epithelial cell model from dairy goats, *Rhaponticum uniflorum* was shown to promote milk protein transcription and enhance lactose synthesis [9]. SCC is the core indicator for evaluating breast health and milk quality. A high cell count usually indicates mastitis or tissue damage, and is directly related to microbial contamination of milk and a decline in sensory quality [36]. The significantly reduced SCC observed in both the CHM and FCHM groups’ colostrum demonstrates the potent anti-inflammatory effects of the compound herbal medicine in mitigating postpartum inflammatory responses in sows. The elevated milk fat content observed with FCHM supplementation stems from microbial fermentation rather than the herbal matrix itself. During fermentation, *Limosilactobacillus fermentum* produces short-chain fatty acids (SCFAs), particularly butyrate and propionate, which serve as direct precursors for de novo fatty acid synthesis in mammary epithelial tissue. Additionally, fermentation enhances the bioavailability of lipid-soluble vitamins and other micronutrients that support milk fat synthesis, effects absent in non-fermented CHM [37]. The increased serum UN reflects the improved nitrogen metabolism efficiency resulting from fermentation. Fermentation increases protein digestibility and generates microbial metabolites that optimize amino acid utilization, leading to more active nitrogen turnover to support the heightened metabolic demands of lactation. This represents enhanced metabolic capacity rather than compromised protein status.

During pregnancy, maternal physiological functions and metabolism undergo significant fluctuations, requiring substantial energy expenditure to support various biological processes. This heightened metabolic activity generates excessive reactive oxygen species (ROS) and MDA, leading to metabolic disorders, tissue damage, and cellular apoptosis [38]. The body’s primary antioxidant enzymes, including SOD and GSH-Px, play crucial roles in scavenging ROS and repairing oxidative damage to protect cells [39]. MDA, as a metabolic byproduct of oxidative stress, disrupts cell membrane lipids and serves as a reliable indicator of oxidative damage in livestock. In the present study, both CHM and FCHM groups demonstrated significantly elevated serum SOD concentrations in sows and piglets, along with markedly reduced MDA levels in sows, indicating enhanced antioxidant capacity in both mothers and offspring. Similarly, polysaccharides from *Ligusticum chuanxiong* exhibit potent antioxidant and free radical-scavenging properties [40]. Furthermore, *Rhaponticum uniflorum* has been shown to upregulate antioxidant gene expression and mitigate damage to mammary epithelial cells [41]. Additional evidence comes from studies demonstrating that *Limosilactobacillus fermentum ZC529* fermentation enhances T-AOC in macadamia nut husks. In Drosophila models under oxidative stress, aqueous extracts of fermented macadamia nut husks significantly improved survival rates and T-AOC activity, while reducing MDA levels, demonstrating remarkable antioxidant effects [15]. Our results corroborate these findings, showing that *Limosilactobacillus fermentum ZC529* fermentation enhances T-AOC capacity in sows.

The peripartum period places sows in a state of extreme catabolism, predisposing them to postpartum complications, including mastitis, metritis, and agalactia syndrome, which are characterized by elevated pro-inflammatory cytokine levels [42]. Serum immunoglobulins and pro-inflammatory cytokine profiles serve as key indicators of sow health status. In the present study, dietary supplementation with FCHM significantly increased serum IgG levels in sows, an effect potentially attributable to the active components in *Ligusticum chuanxiong*. Previous studies have demonstrated that ligustrazine can markedly upregulated the expression of antimicrobial peptides and reduced inflammatory cytokine levels [43]. Furthermore, ligustrazine exhibits significant antimicrobial activity against pathogens, such as Staphylococcus aureus, *Escherichia coli*, and *Salmonella*, and has been shown to reduce *Salmonella* Typhimurium load and inflammatory responses in broilers, thereby promoting growth and alleviating infection [44]. *Vaccaria segetalis* extracts have also been reported to increase serum immunoglobulins in sows, with particularly pronounced effects during early lactation [6]. Compared to the non-fermented CHM group, the FCHM group showed significantly higher serum IgA and IgM levels, indicating that fermentation enhances the bioavailability of active components and improves their absorption and utilization, especially with regard to antimicrobial and anti-inflammatory effects. Lactic acid bacteria can increase the content of antioxidant compounds in fermentation products through biotransformation. For example, fermenting soybeans with Lactobacillus casei increases phenolic acid and isoflavone content, significantly improving the antioxidant activity of whole-soybean flour [7]. The FCHM group exhibited significantly reduced serum IL-1α and IL-6 levels following herbal supplementation. As IL-1α is released during endometritis and mastitis, and IL-6 primarily participates in postpartum tissue repair, these reductions suggest that, while control group sows experienced acute inflammation or tissue damage, the herbal-supplemented sows maintained better health status post farrowing. This anti-inflammatory effect may be mediated through multiple pathways, as evidenced by studies showing that *Rhaponticum uniflorum* extracts can suppress NF-κB activation and inflammatory cytokine production [45], while its ethanol extracts alleviate inflammation via the Nrf2/HO-1 signaling pathway [46]. The current results further validate this mechanism and highlight the potential of fermented herbal formulations in optimizing peripartum sow health and productivity through integrated immunomodulatory, anti-inflammatory, and antimicrobial actions.

The reproductive physiology of sows depends critically on the dynamic balance of serum E2, Prog, and PRL to maintain pregnancy, support fetal growth, and meet postpartum lactation requirements. During lactation, elevated PRL levels inhibit the hypothalamic–pituitary–gonadal axis, thereby delaying follicular development and E2 secretion [47]. Our study demonstrates that the FCHM effectively modulates this endocrine system through integrated mechanisms. This multi-target effect arises from the synergistic actions of various herbal components. *Vaccaria segetalis* stimulates mammary gland development and modulates lactogenic hormone metabolism, ultimately enhancing milk production [6]. Notably, dibutyl phthalate derived from *Vaccaria segetalis* exhibits both estrogenic and prolactin-like activities, directly enhancing milk synthesis capability in bovine mammary epithelial cells [28]. Furthermore, studies have demonstrated that high-dose *Tetrapanax papyrifer* extract upregulates the expression of three key lactogenic hormone receptors in murine models, thereby promoting hormonal secretion [7]. The fermented preparation’s effects on multiple lactogenic hormones underscore its potential as an effective, multi-targeted supplement for enhancing sow lactation performance through physiological mechanisms that maintain natural hormonal balance.

Non-targeted metabolomics analysis of sow colostrum has revealed significant alterations in endogenous metabolites following supplementation with FCHM, providing novel insights into the mechanisms by which herbal components enhance lactation performance. Our metabolomic investigation revealed that the FCHM induced significant accumulation of differential metabolites with antimicrobial, anti-inflammatory, and immunomodulatory functions in sow colostrum. Edasalonexent is an orally bioavailable small-molecule inhibitor of NF-κB that effectively attenuates inflammatory responses and suppresses fibrosis [48]. Valeric acid directly modulates both lipid and glucose metabolism through multiple pathways [49]. Particularly noteworthy is the identification of pregabalin-mediated effects on mammary gland function. Experimental evidence suggests that this compound enhances leptin production; it not only stimulates β-casein gene expression and milk protein synthesis but also promotes mammary epithelial cell proliferation [50]. An analysis of sow colostrum revealed that feeding with FCHM significantly enriched metabolic pathways linked to lactogenic hormone regulation and milk synthesis. Compared to CON, the FCHM group exhibited marked alterations in key KEGG pathways, including OT and PRL signaling, galactose/starch/sucrose metabolism, aminoacyl-tRNA biosynthesis, fatty acid biosynthesis, pantothenate and CoA biosynthesis, oxidative phosphorylation, and ABC transporters. These modifications collectively enhance lactation performance. OT signaling, via G protein-coupled receptors on myoepithelial cells, triggers phospholipase C-IP3-mediated calcium transients essential for milk ejection [51]. PRL, primarily regulated by hypothalamic dopaminergic inhibition counteracted by cAMP pathway activation (e.g., via 1-Methylxanthine), activates milk protein gene transcription through JAK2-STAT5 [52]. Energy for synthesis is predominantly (>60%) supplied by mitochondrial oxidative phosphorylation, whose activity correlates with milk production [53]. While these integrated pathways explain FCHM-induced improvements in milk quantity and quality, the specific mechanisms by which fermented herbal components modulate sow milk metabolism require further investigation, highlighting their potential as natural lactogenic supplements in swine production.

These improvements stem from the inherent bioactive compounds in the herbal formula. Both treatments significantly reduced sow weight loss and decreased low-birthweight piglets while increasing average birthweight. They enhanced milk yield and quality by reducing somatic cell count and elevating milk protein and lactose. Antioxidant capacity improved markedly through decreased MDA and increased SOD activity. Immune function was enhanced via higher serum IgG, reduced pro-inflammatory cytokines IL-1α and IL-6, and improved reproductive hormone balance as shown by decreased E2 alongside increased Prog. In piglets, both treatments increased weaning litter weight, average weaning weight, and serum SOD activity and IgG levels. The fermentation process with *Limosilactobacillus fermentum ZC529* provided additional significant benefits beyond raw herbs. FCHM uniquely improved birth litter weight, elevated postpartum feed intake, and enhanced milk composition by increasing milk fat and urea nitrogen content. It further boosted sow antioxidant status with elevated T-AOC, enhanced humoral immunity through increased IgM, and specifically elevated prolactin levels, with metabolomic analysis revealing enrichment of the prolactin signaling pathway.

## 5. Conclusions

FCHM with *Limosilactobacillus fermentum ZC529* enhanced its nutritional value and bioactive component bioavailability. Dietary supplementation with FCHM significantly altered colostrum metabolites associated with lactation and milk quality in Ningxiang sows, with differential metabolites enriched in oxytocin-related metabolic pathways. This intervention improved reproductive performance by increasing perinatal feed intake, enhancing antioxidant capacity and immune function, and reducing postpartum inflammatory responses in sows. Additionally, it promoted growth performance and potentially strengthened immune competence in offspring piglets. Future studies should optimize the other livestock and assess long-term feeding safety across multiple reproductive cycles.

## Figures and Tables

**Table 1:** Basal diet composition (air-dry basis).

Item	Gestation	Lactation
Ingredients %		
Corn	61.00	61.40
Soybean meal	6.90	18.60
Wheat feed flour	18.00	5.00
Puffing soybean	0.00	5.00
Soybean hulls	10.00	3.00
Glucose	0.00	2.00
Soybean oil	1.20	1.60
limestone meal	1.00	1.00
NaHCO_3_	0.30	0.85
Salt	0.50	0.45
Choline chloride (60%)	0.10	0.10
Premix ^1^	1.00	1.00
Total	100.00	100.00
Nutrient level		
Digestible Energy ^2^, Kcal/kg	3050	3333
Crude protein ^3^, %	11.51	15.53
Calcium ^3^, %	0.56	0.67
Total phosphorus ^3^, %	0.52	0.57
Lys ^2^, %	0.52	0.81

^1^ Provided per kilogram of complete diets: 9750 IU vitamin A, 3 mg vitamin B1, 7.5 mg vitamin B2, 4.5 mg vitamin B6, 0.03 mg vitamin B12, 3000 IU vitamin D3, 30 g vitamin E, 0.15 mg biotin, 1.5 mg folic acid, 24 mg D-pantothenic acid, 30 mg nicotinic acid, 16 mg Cu, 120 mg Fe, 65 mg Zn, 30 mg Mn, 1 mg I, 0.30 mg Se, 0.13 mg Cr. ^2^ As calculated value. ^3^ As measured value.

**Table 2:** Changes in nutrients and active components before and after fermentation (air-dry basis).

Item ^1^	Content Before Fermentation	Content After Fermentation	*p*-Value
CP, %	12.03 ± 0.41 ^b^	13.66 ± 0.36 ^a^	0.039
EE, %	2.52 ± 0.58	2.55 ± 0.41	0.969
CF, %	12.09 ± 0.18 ^b^	13.58 ± 0.19 ^a^	0.005
Ash, %	5.45 ± 0.18	5.34 ± 0.03	0.583
Total Flavonoids, %	6.82 ± 0.88 ^b^	8.42 ± 0.64 ^a^	0.036
Total Polysaccharides, %	19.52 ± 1.15 ^b^	26.2 ± 1.85 ^a^	0.005
Total Saponins, %	1.88 ± 0.36	1.76 ± 0.22	0.623
Total Alkaloids, %	9.81 ± 0.22	10.34 ± 0.6	0.217

^1^ Results are presented as means with SEM (*n* = 6). CP, Crude Protein; EE, Ether Extract; CF, Crude Fiber. ^a, b^ Values in the same row with different letter are significantly different (*p* < 0.05).

**Table 3:** Effects of dietary FCHM supplementation on the reproductive performance of sows.

Item ^1^	CON Group	CHM Group	FCHM Group	*p*-Value
Initial weight of sows, kg	101.17 ± 1.23	100.76 ± 0.61	101.61 ± 2.57	0.937
Last weight of sows, kg	94.06 ± 1.14	91.3 ± 0.83	91.73 ± 2.01	0.346
Sow weight loss, kg	7.11 ±0.35 ^b^	9.45 ±0.67 ^a^	9.88 ± 0.68 ^a^	0.004
Total piglets born	8.30 ± 0.33	8.11 ± 0.35	9.00 ± 0.42	0.225
Live-born piglets	7.80 ± 0.36	7.78 ± 0.43	8.80 ± 0.47	0.163
Stillborn piglets	0.40 ± 0.16	0.33 ± 0.17	0.20 ± 0.13	0.644
High-birthweight piglets ^2^	6.60 ± 0.45 ^b^	7.11 ± 0.39 ^ab^	8.30 ± 0.45 ^a^	0.027
Low-birthweight piglets ^3^	1.30 ± 0.15 ^a^	0.67 ± 0.24 ^b^	0.40 ± 0.16 ^b^	0.005
Total litter birthweight, kg	6.58 ± 0.32 ^b^	6.74 ± 0.32 ^b^	7.91 ± 0.44 ^a^	0.031
Average birthweight, kg	0.81 ± 0.01 ^b^	0.87 ± 0.02 ^a^	0.89 ± 0.02 ^a^	0.02
Total born process time/min	113.10 ± 7.95	100.67 ± 6.94	110.9 ± 7.64	0.486
Average born process time/min	13.60 ± 0.78	12.37 ± 0.61	12.28 ± 0.48	0.274
Milk yield, kg	56.04 ± 3.00 ^b^	75.64 ± 2.78 ^a^	88.18 ± 8.39 ^a^	0.001

^1^ Results are presented as means with SEM (*n* = 10). CON, basal diet; CHM, basal diet + 2% Chinese herbal medicine; FCHM, basal diet + 2% fermented Chinese herbal medicine. ^a, b^ Values in the same row with different letter are significantly different (*p* < 0.05). ^2^ High-birthweight piglets were defined as those with birthweight > 0.8 kg. ^3^ Low-birthweight piglets were defined as those with birthweight ≤ 0.8 kg.

**Table 4:** Effects of dietary FCHM supplementation on feed intake of sows.

Item ^1^	CON Group	CHM Group	FCHM Group	*p*-Value
The week before farrowing, kg	10.82 ± 0.05 ^b^	10.52 ± 0.08 ^b^	11.8 ± 0.18 ^a^	<0.001
The first week after farrowing, kg	4.67 ± 0.84 ^c^	6.12 ± 0.25 ^b^	8.14 ± 0.27 ^a^	<0.001
The second week after farrowing, kg	10.39 ± 0.16 ^b^	10.51 ± 0.34 ^b^	14.24 ± 0.39 ^a^	<0.001
The third week after farrowing, kg	15.31 ± 0.47 ^c^	18.54 ± 0.23 ^b^	19.96 ± 0.20 ^a^	<0.001

^1^ Results are presented as means with SEM (*n* = 10). CON, basal diet; CHM, basal diet + 2% Chinese herbal medicine; FCHM, basal diet + 2% fermented Chinese herbal medicine. ^a, b, c^ Values in the same row with different letter are significantly different (*p* < 0.05).

**Table 5:** Effects of dietary FCHM supplementation on the growth performance of piglets.

Item ^1^	CON Group	CHM Group	FCHM Group	*p*-Value
Lactation survival rate, %	90.46 ± 2.90	90.71 ± 3.42	95.99 ± 2.01	0.323
Lactation diarrhea rate, %	5.03 ± 0.19	5.24 ± 0.46	4.68 ± 0.38	0.569
Weaning litter weight, kg	20.59 ± 0.87 ^b^	25.66 ± 0.88 ^a^	27.54 ± 1.18 ^a^	<0.001
Weaning individual mean weight, kg	2.76 ± 0.07 ^b^	3.40 ± 0.10 ^a^	3.51 ± 0.10 ^a^	<0.001
No. of weaned piglets	7.5 ± 0.37	7.33 ± 0.37	8.56 ± 0.52	0.117

^1^ Results are presented as means with SEM (*n* = 10). CON, basal diet; CHM, basal diet + 2% Chinese herbal medicine; FCHM, basal diet + 2% fermented Chinese herbal medicine. ^a, b^ Values in the same row with different letter are significantly different (*p* < 0.05).

**Table 6:** Effects of dietary FCHM supplementation on colostrum composition of sows.

Item ^1^	CON Group	CHM Group	FCHM Group	*p*-Value
SCC, ×10^3^ cells/mL	1771 ± 339.91 ^a^	657 ± 60.33 ^b^	1087 ± 160.51 ^b^	0.007
Milk fat rate, %	4.07 ± 0.32 ^b^	4.21 ± 0.49 ^b^	5.48 ± 0.11 ^a^	0.035
Milk protein rate, %	16.98 ± 0.23 ^b^	18.55 ± 0.62 ^a^	18.99 ± 0.55 ^a^	0.02
Lactose rate, %	3.36 ± 0.13 ^b^	3.86 ± 0.10 ^a^	3.98 ± 0.04 ^a^	0.001
NFS, %	26.28 ± 0.94	25.02 ± 0.60	25.83 ± 0.61	0.486
UN, mg/dL	43.08 ± 2.57 ^b^	45.80 ± 2.01 ^b^	54.67 ± 0.95 ^a^	0.002

^1^ Results are presented as means with SEM (*n* = 6). CON, basal diet; CHM, basal diet + 2% Chinese herbal medicine; FCHM, basal diet + 2% fermented Chinese herbal medicine. SCC, Somatic cell count; NFS, Non-fat solids content; UN, Urea nitrogen. ^a, b^ Values in the same row with different letter are significantly different (*p* < 0.05).

**Table 7:** Effects of dietary FCHM supplementation on serum biochemical indices of sows and piglets.

Item ^1^	CON Group	CHM Group	FCHM Group	*p*-Value
Sow				
MDA, nmol/mL	5.11 ± 0.23 ^a^	3.77 ± 0.18 ^b^	4.36 ± 0.25 ^b^	0.002
T-AOC, U/mL	0.87 ± 0.02 ^b^	0.89 ± 0.01 ^b^	0.94 ± 0.01 ^a^	0.010
GSH-Px, μmol/mL	102.23 ± 4.99	113.43 ± 12.79	100.71 ± 6.35	0.520
SOD, U/mL	219.05 ± 10.22 ^b^	405.64 ± 10.5 ^a^	422.87 ± 13.37 ^a^	<0.001
IgA, μg/mL	54.88 ± 1.79 ^b^	59.23 ± 2.11 ^ab^	63.30 ± 3.05 ^a^	0.024
IgM, μg/mL	203.25 ± 46.8 ^b^	211.27 ± 24.34 ^b^	348.03 ± 18.32 ^a^	0.014
IgG, mg/mL	2.00 ± 1.62 ^b^	6.29 ± 1.80 ^a^	7.02 ± 1.48 ^a^	0.040
TNF-α, pg/mL	15.64 ± 0.25	15.78 ± 0.67	15.07 ± 0.45	0.590
IL-1α, pg/mL	156.25 ± 7.8 ^a^	41.03 ± 5.95 ^b^	54.81 ± 10.46 ^b^	<0.001
IL-6, pg/mL	120.29 ±13.29 ^a^	69.81 ± 17.32 ^b^	68.12 ± 13.76 ^b^	0.038
E2, ng/mL	1442.93 ± 76.41 ^a^	822.55 ± 181.31 ^b^	442.26 ± 101.2 ^b^	0.002
Prog, ng/mL	116.99 ± 9.27 ^b^	215.56 ± 10.53 ^a^	214.08 ± 46.67 ^a^	0.028
PRL, ng/mL	0.92 ± 0.06 ^b^	1.08 ± 0.13 ^b^	1.66 ± 0.20 ^a^	0.012
Piglets				
MDA, nmol/mL	4.09 ± 0.11	3.50 ± 0.35	3.61 ± 0.24	0.242
T-AOC, U/mL	0.87 ± 0.03	0.97 ± 0.02	0.85 ± 0.10	0.372
GSH-Px, μmol/mL	99.30 ± 6.93	94.32 ± 6.08	93.24 ± 4.79	0.752
SOD, U/mL	108.22 ± 28.07 ^b^	252.87 ± 44.40 ^a^	237.19 ± 15.25 ^a^	0.015
IgA, μg/mL	53.26 ± 1.75	57.98 ± 2.20	57.66 ± 2.72	0.286
IgM, μg/mL	63.03 ± 11.4	90.81 ± 24.88	90.12 ± 9.94	0.437
IgG, mg/mL	1.64 ± 0.53 ^b^	2.42 ± 0.27 ^a^	2.47 ± 0.26 ^a^	0.087
TNF-α, pg/mL	15.48 ± 0.41	15.36 ± 0.46	15.12 ± 0.30	0.890
IL-1α, pg/mL	29.32 ± 2.17	29.31 ± 1.61	39.50 ± 6.51	0.148
IL-6, pg/mL	173.32 ± 25.06	176 ± 15.00	133.78 ± 20.44	0.290

^1^ Results are presented as means with SEM (*n* = 6). CON, basal diet; CHM, basal diet + 2% Chinese herbal medicine; FCHM, basal diet + 2% fermented Chinese herbal medicine. MDA, Malondialdehyde; T-AOC, Total antioxidant capacity; GSH-Px, Glutathione peroxidase; SOD, Superoxide dismutase; IgA, Immunoglobulin; IgM, Immunoglobulin M; IgG, Immunoglobulin G; IL-1α, Interleukin-1α; IL-6, Interleukin-6; TNF-α, Tumor necrosis factor-α; E2, Estradiol; Prog, Progesterone; PRL, Prolactin. ^a, b^ Values in the same row with different letter are significantly different (*p* < 0.05), For piglet IgG, superscripts indicate a tendency (0.05 < *p* < 0.10).

**Table 8:** Effects of FCHM on non-targeted differential metabolites in sow colostrum.

Compounds	Regulation	log_2_FC	*p*-Value	VIP	MODE
2-Chloro-N,N-dimethylethanaminium	Down	−5.2055	0.0002	2.450	NEG
4-Methoxyphenol sulfate	Down	−5.0016	0.0011	2.433	NEG
P-Cresol sulfate	Up	7.8237	0.0003	2.424	NEG
Paracetamol sulfate	Down	−6.6904	0.0021	2.410	NEG
Edgeworoside A	Up	3.6893	<0.0001	2.401	NEG
Asperitaconic acid C	Down	−5.6892	0.0019	2.388	NEG
1-Methylxanthine	Down	−0.6340	<0.0001	2.367	POS
N-Cyclohexyl-2-benzothiazolesulfenamide	Down	−5.4209	0.0020	2.365	NEG
Ethyl 2-bromopropanoate	Up	0.9955	<0.0001	2.357	NEG
Fasciculic acid C	Up	2.9250	0.0002	2.351	NEG
Tert-Butyl 2,4-dibromobutyrate	Up	1.3334	<0.0001	2.342	NEG
Pregabalin	Up	4.6286	0.0021	2.319	NEG
4-Ethylphenylsulfate	Up	6.8102	0.0318	2.318	NEG
Edasalonexent	Up	6.9704	0.0040	2.300	POS
Pyridine-2,6-dicarboxamide	Down	−0.6660	0.0001	2.274	POS
11-amino-undecanoic acid	Up	3.9976	0.0031	2.246	NEG
Methyleugenol	Down	−0.8022	0.0006	2.228	POS
Valeric acid	Up	3.6479	0.0028	2.216	NEG
PG 28:5	Down	−1.9045	0.0011	2.199	NEG
Phenyllithium	Up	3.1115	0.0033	2.172	POS

FC stands for fold change, and the selection conditions for the metabolites shown in the table are FC < 1, *p* < 0.05, VIP > 1. VIP = variable importance in the projection; NEG = negative ion mode; POS = positive ion mode.

## Data Availability

The original contributions presented in the study are included in the article; further inquiries can be directed to the corresponding authors.

## Abbreviations

The following abbreviations are used in this manuscript:

CHM	Chinese herbal medicine
FCHM	Fermented Chinese herbal medicine
MDA	Malondialdehyde
SOD	Superoxide dismutase
Prog	Progesterone
E2	Estradiol
T-AOC	Total antioxidant capacity
PRL	Prolactin
Ig A	Immunoglobulin A
Ig M	Immunoglobulin M
Ig G	Immunoglobulin G
IL-1α	Interleukin-1α
IL-6	Interleukin-6
TNF-α	Tumor necrosis factor-α
GSH-Px	Glutathione peroxidase
SCC	Somatic cell count
NFS	Non-fat solids
UN	Urea nitrogen
CP	Crude Protein
EE	Ether Extract
CF	Crude Fiber
OT	Oxytocin
ROS	Reactive oxygen species
VIP	Variable importance in the projection
